# The potential role of large language models in addressing phenoconversion in pharmacogenomics

**DOI:** 10.3389/fgene.2026.1987469

**Published:** 2026-10-21

**Authors:** Augusto García-Agúndez, José A. G. Agúndez

**Affiliations:** 1 Division of Rheumatology, University of California San Francisco, San Francisco, CA, United States; 2 University Institute of Molecular Pathology Biomarkers, Universidad de Extremadura, Cáceres, Spain

**Keywords:** drug-drug-gene interactions, electronic health records, large language models, pharmacogenomics, phenoconversion, precision pharmacotherapy

## Abstract

Pharmacogenomics (PGx) is a cornerstone of precision medicine, enabling individualized drug therapy by identifying genetic factors that influence drug disposition and response. However, genotype-based predictions frequently fail to reflect actual drug-metabolizing capacity because enzyme activity can be modified by non-genetic factors, including concomitant medications, inflammation, organ dysfunction, aging, smoking, pregnancy, and other environmental influences. Phenoconversion can generate substantial discrepancies between genotype-predicted and observed metabolic phenotypes, contributing to adverse drug reactions, therapeutic failure, and reduced clinical utility of pharmacogenomic testing. The identification of phenoconversion remains challenging because the relevant determinants are often dispersed across multiple sources of information, including medication records, laboratory data, diagnostic information, and unstructured clinical notes within electronic health records. Recent advances in large language models (LLMs) offer new opportunities to address this limitation by retrieving, organizing, and contextualizing heterogeneous clinical and pharmacogenomic information. Beyond identifying drug-drug-gene and drug-gene-environment interactions, LLM-enabled systems may support dynamic phenotype assessment by modeling metabolic capacity as a continuously evolving characteristic rather than a static genotype-derived category and by identifying relevant modifiers even for drugs without actionable pharmacogenomic recommendations. This review examines the biological basis and clinical significance of phenoconversion and discusses the potential role of LLMs in bridging the gap between genetic predisposition and real-world drug response. We highlight the emerging concept of dynamic phenotype models that combine genetic, clinical, and environmental determinants of drug metabolism and may support more precise, context-aware pharmacotherapy.

## Introduction

Precision medicine seeks to tailor healthcare interventions according to the characteristics of each individual, recognizing that genetic, environmental, and physiological factors contribute to marked differences in disease susceptibility and treatment response. Among the various precision-medicine approaches, pharmacogenomics can provide clinically actionable information for selected gene-drug pairs because inherited variation in genes involved in drug disposition and pharmacological targets may influence therapeutic efficacy and the risk of adverse drug reactions. Evidence accumulated over the last two decades has established the clinical relevance of variation in pharmacogenes including *CYP2D6*, *CYP2C19*, *CYP2C9*, *DPYD*, *SLCO1B1*, and *NAT2*. However, implementation remains uneven and is concentrated in particular drugs, specialties, institutions, and healthcare systems (Kabbani et al., 2023). Moreover, genetic variation represents only one source of interindividual variability in drug exposure and response. Precision dosing may also require consideration of non-genetic covariates, measured drug concentrations, organ function, interacting medications, disease-related factors, and individual treatment response (Abad-Santos et al., 2024; Caudle et al., 2014; Agundez and Garcia-Martin, 2026; Minichmayr et al., 2024; Keutzer and Simonsson, 2020).

The translation of pharmacogenomic discoveries into healthcare practice has been driven not only by advances in molecular genetics but also by the establishment of international collaborative initiatives dedicated to the standardization and dissemination of pharmacogenomic knowledge. Organizations such as the Clinical Pharmacogenetics Implementation Consortium (CPIC) and the Dutch Pharmacogenetics Working Group (DPWG) have developed rigorously curated recommendations that assist clinicians in adjusting drug selection and dosage based on a patient’s genetic profile. The contribution of informatics and curation resources that provide the infrastructure required for consistent interpretation of genetic results has been equally important. PharmGKB has become a central repository for pharmacogenomic evidence, integrating information on gene-drug interactions, variant function, clinical annotations, and prescribing guidelines (Gong et al., 2021). In parallel, ClinVar and ClinGen have facilitated the systematic collection, assessment, and sharing of information regarding the clinical significance of genetic variants, thereby improving the reliability and reproducibility of pharmacogenetic reporting (Landrum et al., 2020; Rehm et al., 2015).

Standardized allele nomenclature has also proven essential for the successful implementation of pharmacogenomics. In this regard, the Pharmacogene Variation Consortium (PharmVar) serves as the reference authority for defining and updating pharmacogene haplotypes and star-allele nomenclature, enabling harmonized communication among laboratories, researchers, and healthcare providers (Gaedigk et al., 2018; Papanikolaou et al., 2026; Turner et al., 2023). Recent efforts to refine the classification of highly polymorphic genes, including *NAT2*, *CYP2D6*, and *DPYD*, have highlighted the importance of ongoing curation as new functional and population-genetic data become available (Papanikolaou et al., 2026; Turner et al., 2023). Collectively, these resources and consortia have established an increasingly standardized framework for translating pharmacogenomic evidence into prescribing recommendations. Nevertheless, the availability of curated recommendations does not necessarily result in routine implementation, and the clinical use of pharmacogenomics remains variable across drugs, specialties, institutions, and healthcare systems (Kabbani et al., 2023; Pritchard et al., 2022).

Pharmacogenomic testing is an established component of precision medicine for selected indications, but genotype alone frequently provides an incomplete representation of an individual’s drug-metabolizing capacity. This limitation is consistent with the broader principles of precision dosing, in which genetic information represents one of several patient-specific determinants that may influence pharmacokinetics, pharmacodynamics, efficacy, and toxicity. Model-informed precision dosing provides an established framework for integrating information on drug properties, patient characteristics, prior observations, and mechanistic or population-level models to support individualized dose selection (Darwich et al., 2017; Poweleit et al., 2023). Within this framework, pharmacogenomic information may be incorporated alongside interacting medications, organ function, disease status, therapeutic drug monitoring, and other time-varying covariates (Minichmayr et al., 2024; Mostafa et al., 2022). The potential role of large language models (LLMs) should therefore be understood as complementary to established precision-dosing approaches rather than as an alternative to pharmacometric modeling (Darwich et al., 2017; Polasek et al., 2019; Wicha et al., 2021; Bates et al., 2022). Drug response is determined not only by inherited genetic variation but also by numerous environmental, physiological, and pathological factors that can dynamically modify enzyme activity throughout life. This phenomenon, commonly referred to as phenoconversion, describes the mismatch between genotype-predicted metabolizer status and the patient’s current functional metabolic phenotype. Consequently, individuals genetically classified as normal metabolizers may function phenotypically as intermediate or poor metabolizers, whereas enzyme induction can occasionally produce the opposite effect. Recent evidence has identified phenoconversion as an important limitation of genotype-only predictions, and several authors have proposed integrating pharmacogenomic results with clinical and pharmacological information to support precision dosing (Mostafa et al., 2022; Nahid and Johnson, 2022; Scodellaro et al., 2026; Klomp et al., 2020; Shah and Smith, 2015; Cicali et al., 2021; Preskorn et al., 2013; Stingl and Viviani, 2025). The most thoroughly characterized examples involve cytochrome P450 enzymes, particularly *CYP2D6, CYP2C19*, whereas CYP3A-mediated activity may also be substantially modified by non-genetic factors. Strong CYP2D6 inhibitors, including paroxetine, fluoxetine, bupropion, and quinidine, can reduce enzyme activity to levels comparable to those observed in poor metabolizers, thereby altering drug exposure and increasing the risk of adverse drug reactions. Similar effects have been reported for CYP2C19 and CYP3A enzymes, whose activity may be modified by concomitant medications, inflammatory conditions, liver dysfunction, pregnancy, smoking, aging, and other environmental influences (Nahid and Johnson, 2022; Scodellaro et al., 2026; Scherf-Clavel et al., 2024). Importantly, inflammation-mediated suppression of CYP expression has emerged as a major contributor to phenoconversion, particularly in patients with infectious diseases, autoimmune disorders, cancer, or critical illness (Scodellaro et al., 2026). Consequently, metabolic capacity should be viewed as a dynamic phenotype rather than a fixed genetic trait.

Mounting evidence suggests that phenoconversion is remarkably common in real-world clinical settings, particularly among patients exposed to polypharmacy (Patel et al., 2024). Large cohort studies have demonstrated that consideration of concomitant medications and other non-genetic factors substantially increases the proportion of patients with clinically actionable pharmacogenomic phenotypes compared with genotype-based prediction alone (Patel et al., 2024). These observations have important therapeutic implications because discrepancies between genotype-predicted and actual metabolic status may result in unexpected toxicity when drug metabolism is inhibited or in therapeutic failure when metabolic pathways are induced. Such effects are especially relevant for medications with narrow therapeutic indices and for drugs whose efficacy depends on metabolic activation, including tamoxifen, codeine, tramadol, and numerous psychotropic agents.

Recognition of phenoconversion has contributed to a broader conceptual shift within pharmacogenomics. Rather than relying exclusively on static genotype-derived phenotype assignments, there is growing interest in integrating drug-drug-gene interactions, drug-gene-environment interactions, comorbidity profiles, and biomarkers of physiological status into pharmacogenomic interpretation. This broader approach overlaps substantially with model-informed precision dosing and related pharmacometric methods, which seek to combine multiple patient-specific determinants with quantitative models of pharmacokinetics and, where sufficiently characterized, pharmacodynamics (Darwich et al., 2017; Wicha et al., 2021; Bates et al., 2022). Dynamic phenotype assessment should therefore not be presented as conceptually separate from the established precision-dosing field (Polasek et al., 2019; Klomp et al., 2020; Porrogi, 2026). The distinctive contribution proposed here is the use of LLMs to retrieve and contextualize relevant time-varying covariates, particularly when those covariates are dispersed across structured and unstructured clinical records. This transition reflects the growing recognition that drug-metabolizing capacity is a multifactorial phenotype arising from interactions between inherited genetic variation and a continuously evolving clinical environment. Recent pharmacogenomic guidance documents, reviews, and clinical studies have increasingly emphasized the potential importance of phenoconversion when translating genetic information into prescribing decisions (Agundez and Garcia-Martin, 2026; Mostafa et al., 2022; Nahid and Johnson, 2022; Klomp et al., 2020; Scherf-Clavel et al., 2024; Scherf-Clavel et al., 2023; Cameron et al., 2026; Thottunkal et al., 2025).

The challenge, however, lies in the practical implementation of such an approach. Most determinants of phenoconversion are dispersed across multiple components of the electronic health record (EHR), including medication lists, laboratory measurements, diagnostic codes, clinical notes, and imaging reports. Furthermore, important information such as over-the-counter drug use, smoking cessation, inflammatory disease flares, or temporary physiological conditions is frequently documented only in unstructured clinical narratives. As a result, clinicians are often required to integrate large amounts of fragmented information within limited consultation times, making systematic assessment of phenoconversion difficult in routine practice. Similar implementation barriers have been recognized in precision dosing more generally. Even when relevant genetic, pharmacokinetic, and clinical information is available, its use may be limited by the time, expertise, and infrastructure required to transform multiple sources of information into an actionable prescribing recommendation. Clinical decision-support systems must therefore reduce, rather than add to, the cognitive and operational burden placed on prescribers (Agundez and Garcia-Martin, 2026; Darwich et al., 2017; Poweleit et al., 2023; Polasek et al., 2019; Dibbets et al., 2025).

Concurrently, healthcare systems have experienced an unprecedented expansion in the volume and complexity of digital clinical information. Modern EHRs increasingly incorporate longitudinal prescribing data, laboratory results, diagnostic information, genomic findings, and pharmacogenomic reports. Recent advances in artificial intelligence, particularly LLMs, have demonstrated considerable potential to extract clinically relevant information from heterogeneous datasets, summarize complex patient histories, identify drug-drug interactions, and support clinical decision-making through context-aware reasoning (Du et al., 2026). These capabilities are especially attractive in pharmacogenomics because the factors driving phenoconversion are often distributed across structured and unstructured data sources, creating challenges for traditional rule-based clinical decision-support systems, particularly when relevant information is embedded in unstructured text or requires temporal contextualization (Knorr et al., 2026).

The convergence of pharmacogenomics, real-world clinical data, pharmacometric modelling, and artificial intelligence offers an opportunity to improve the identification and interpretation of patient-specific factors that influence drug metabolism and exposure. This approach should build on established model-informed precision-dosing methods rather than replace them (Darwich et al., 2017). LLMs may help extract and temporally organize information from heterogeneous clinical sources, whereas validated pharmacokinetic and pharmacodynamic models would remain necessary to translate those inputs into quantitative predictions. Future clinical decision-support platforms may move beyond static pharmacogenomic interpretation by generating dynamic representations of metabolic capacity that integrate genetic variation with medication exposure, laboratory findings, inflammatory markers, comorbidities, lifestyle factors, and other environmental determinants. Such systems could support more context-aware estimates of drug-metabolizing capacity and, when combined with validated drug-specific pharmacokinetic and pharmacodynamic models, may contribute to improved predictions of exposure, efficacy, or toxicity. Recent advances in multimodal artificial intelligence (AI), together with emerging applications of AI-driven multi-omics integration, suggest that these technologies may facilitate the transition from a predominantly gene-centered paradigm toward a truly individualized and phenotype-centered model of precision pharmacotherapy (Zack et al., 2025a).

## Large language models as clinical knowledge integrators

Identifying potential phenoconversion is partly a clinical knowledge-integration problem. Genotype provides a baseline prediction of metabolic phenotype, but determining whether that prediction reflects a patient’s current metabolic state requires consideration of additional factors scattered across the EHR. In this sense, phenoconversion detection is a highly complex inference problem: relevant signals may be distributed across the clinical record, and the factors or combinations of factors responsible for altered metabolic activity may not always be known *a priori*. LLMs may be particularly well-suited to this type of problem (Garcia-Agundez et al., 2026). Their potential value lies not only in extracting individual clinical variables but also in linking temporally related information distributed across the EHR and presenting it in the context of the patient’s pharmacogenomic profile (Guevara et al., 2024). In this role, an LLM could bring together otherwise disconnected observations to identify evidence that a genotype-predicted phenotype may no longer reflect the patient’s likely functional metabolic state.

For example, a patient may have a pharmacogenomic result indicating CYP2D6 normal metabolizer status while simultaneously receiving a CYP2D6 inhibitor and experiencing an inflammatory illness associated with reduced CYP expression. These observations may be documented in different parts of the EHR and, individually, may not indicate phenoconversion. An LLM-assisted system could connect these observations, assess their temporal relationship, and flag an increased likelihood that the genotype-predicted CYP2D6 phenotype no longer reflects current enzyme activity, provided that the system has access to validated knowledge sources. This output would indicate potential phenoconversion rather than confirm that phenoconversion had occurred (Wittwer et al., 2025).

In practice, this would require identifying phenoconversion-relevant variables wherever they occur in the clinical record. Medication exposures may be documented in medication lists, progress notes, discharge summaries, outside records, patient messages, or pharmacy data and may include recently started or discontinued medications, dose changes, over-the-counter medications, and supplements. Environmental and lifestyle exposures, such as smoking or recent smoking cessation, may appear only in narrative documentation. Similarly, evidence of infection, inflammation, cancer, or autoimmune disease may be distributed across laboratory results, diagnoses, and clinical notes. LLMs could extract these signals while preserving clinically relevant context, including whether an exposure is current or historical and when it occurred relative to changes in treatment or clinical response.

The temporal dimension is particularly important because phenoconversion is dynamic (Faber and Fuhr, 2004). The presence of an inhibitor, inflammatory illness, or environmental exposure at any point in a longitudinal record does not establish that it affected metabolism at the clinically relevant time. An LLM could help reconstruct when exposures began or ended and relate them to drug concentrations, adverse effects, therapeutic response, or other evidence of altered metabolism. As new information enters the record, the same approach could support reassessment of whether the patient’s current clinical context remains consistent with the phenotype predicted by genotype.

This approach may also extend beyond searching for predefined causes of phenoconversion. By reasoning across multiple sources of clinical information, LLMs could help identify less obvious combinations of documented factors for subsequent evaluation. Such outputs should be treated as hypotheses requiring confirmation through pharmacological evidence, independent phenotypic measurements, or appropriately designed clinical studies. In this sense, the role of the LLM as a clinical knowledge integrator is to identify and connect clinically relevant signals within a large and evolving record, providing a basis for detecting potential phenoconversion.

## Building dynamic phenotype models

One potential application of LLMs in pharmacogenomics is to provide clinical inputs for dynamic phenotype models. The broader concept of dynamically updating individualized predictions using patient-specific covariates is not new and is central to model-informed precision dosing, population pharmacokinetics, Bayesian forecasting, physiologically based pharmacokinetic modelling, and related pharmacometric approaches (Darwich et al., 2017). Current pharmacogenomic reports generally assign patients to discrete genotype-predicted categories, such as poor, intermediate, normal, or ultrarapid metabolizer status. Although these categories provide clinically valuable information, they do not fully capture the continuous and time-varying nature of enzyme activity.

The proposed role of LLMs is not to replace these established quantitative methods. Rather, LLMs may facilitate the extraction of relevant covariates from heterogeneous clinical records, determine whether an exposure or clinical condition was present at the relevant time, identify missing information, and convert narrative observations into structured inputs. Quantitative estimation of metabolic activity, drug exposure, or dose requirements should remain grounded in validated pharmacokinetic, physiologically based, Bayesian, or other model-informed precision-dosing approaches.

Future clinical decision-support systems may combine LLM-supported clinical information extraction with pharmacokinetic models, physiologically based models, Bayesian forecasting, knowledge graphs, and other validated quantitative methods to generate updated estimates of metabolic activity or drug exposure (Polasek et al., 2019). Instead of assigning a single static phenotype, these systems could integrate pharmacogenomic results with information on concomitant medications, inflammatory status, organ function, lifestyle factors, and other determinants of drug metabolism to produce probabilistic assessments of current metabolic capacity. For example, a patient genetically predicted to have normal CYP2D6 activity who is simultaneously receiving a strong CYP2D6 inhibitor and experiencing an inflammatory condition may have a substantially reduced likelihood of functioning as a normal metabolizer. Rather than assigning a fixed category, a dynamic model could estimate the probability that the individual is currently expressing a phenotype closer to that of a poor metabolizer. These estimates would remain predictions unless supported by an independent phenotypic measure, such as a validated probe-drug test, therapeutic drug monitoring, an endogenous biomarker, or another validated measure of enzyme activity. An LLM may identify factors associated with possible phenotype modification, but it cannot by itself establish that phenoconversion has occurred.

Such an approach would extend static pharmacogenomic classification toward continuously updated estimates of metabolic capacity and drug exposure. Prediction of pharmacodynamic or clinical response is more challenging and would additionally require validated drug-specific relationships among exposure, target engagement, efficacy, and toxicity (Cook et al., 2014; Daryaee et al., 2017; Fernandez-Metzler et al., 2026). Accordingly, improved prediction of metabolic activity or pharmacokinetics should not be assumed to provide equally reliable prediction of pharmacodynamic response. Patients with similar drug exposure may have different concentration-response relationships because of variability in disease biology, target sensitivity, baseline risk, tolerance, and other pharmacodynamic determinants (Turner et al., 2015; Olofsen et al., 2026; Oni-Orisan et al., 2021). Rather than assigning a single metabolizer category, future systems could estimate the likelihood that a patient is currently functioning within a given metabolic range and adapt those estimates as new clinical information becomes available. Within this framework, the role of the LLM would extend beyond information extraction, encompassing the integration of diverse clinical data, the interpretation of complex biological interactions, and the communication of clinically relevant insights in a transparent and understandable manner. Ultimately, dynamic phenotype models may help bridge the gap between genotype-predicted and real-world metabolic function, thereby supporting more precise and context-aware pharmacotherapy.

These capabilities may be particularly valuable in primary care and other non-specialized settings, where clinicians routinely manage complex patients receiving multiple medications. By transforming fragmented clinical information into structured and temporally contextualized inputs, AI-enabled decision-support systems could facilitate the integration of pharmacogenomic information into broader precision-dosing workflows. Their clinical value would depend on the availability of validated quantitative models, clear uncertainty estimates, and drug-specific evidence linking the identified covariates to dosing or treatment decisions (Darwich et al., 2017; Polasek et al., 2019; Patel et al., 2024; Du et al., 2026).

## Challenges and limitations

Despite the potential value of LLMs in phenoconversion-aware clinical decision support, several interconnected scientific, technical, regulatory, and operational challenges must be addressed before these systems can be adopted safely and reliably in routine healthcare.

At the most fundamental level, the clinical utility of LLM-based systems depends on both the reliability of the models themselves and the quality of the information available to them. LLMs may generate incorrect statements, misinterpret clinical information, or produce recommendations that are not adequately supported by available evidence. This limitation is not unique to LLM-based approaches. Model-informed precision dosing also depends on adequately characterized relationships among patient covariates, metabolic activity, pharmacokinetics, pharmacodynamics, and clinical outcomes. Computational sophistication cannot compensate for the absence of reliable quantitative information on these relationships (Minichmayr et al., 2024; Keizer et al., 2018). In pharmacogenomics, even isolated errors can have significant clinical consequences, potentially leading to inappropriate prescribing decisions, adverse drug reactions, or therapeutic failure. This risk is particularly relevant when models are required to synthesize information from multiple sources and infer complex drug-drug-gene interactions. Beyond factual hallucinations, errors in temporal reasoning or interpretation of drug-drug-gene interactions may be particularly problematic because phenoconversion depends heavily on the timing and combined effects of multiple clinical factors. Robust validation using real-world clinical datasets, continuous performance monitoring, periodic recalibration, and mandatory human oversight would likely be essential components of any clinically deployed system (Zack et al., 2025b). Furthermore, AI-generated recommendations should always be accompanied by supporting quotations, citations, and explicit documentation of the clinical factors contributing to the prediction, enabling clinicians to verify the source information, assumptions, and evidence underlying the output.

Even a highly reliable model, however, will remain dependent on the completeness and accuracy of the clinical data it receives. Clinical records frequently contain missing, outdated, or inconsistent information. Medication lists may not include over-the-counter drugs, herbal products, dietary supplements, or recently discontinued therapies, all of which may contribute to phenoconversion. Similarly, lifestyle factors such as smoking, alcohol consumption, and medication adherence are often incompletely documented. Because pharmacogenomic knowledge and prescribing guidelines evolve continuously, AI systems will also require mechanisms for regular updating and revalidation to ensure alignment with current evidence. Also, because pharmacogenomic interpretation relies heavily on contextual information, missing data may substantially reduce prediction accuracy and confidence. Clinical LLM pipelines should therefore incorporate mechanisms to identify data gaps, estimate uncertainty, and alert users when insufficient information is available to support reliable recommendations (Kompa et al., 2021).

These limitations in model reliability and clinical data are compounded by gaps in the underlying scientific understanding of phenoconversion itself. Although numerous contributors have been identified, including drug interactions, inflammation, aging, pregnancy, liver disease, and environmental exposures, quantitative estimates of their individual and combined effects are often lacking. In many situations, the magnitude of phenotype modification cannot be predicted with sufficient precision to support formal dosing recommendations. Consequently, even perfectly functioning AI systems would remain constrained by gaps in the underlying scientific knowledge. Improved mechanistic studies, pharmacokinetic modeling, and prospective clinical investigations will be needed to generate the data required for more accurate phenotype prediction. For many enzymes and environmental modifiers, the magnitude and duration of phenoconversion remain insufficiently characterized, limiting the precision of any prediction model regardless of its computational sophistication. An additional challenge is the lack of widely accepted reference standards for measuring functional metabolic activity in routine clinical practice, which complicates both the development and validation of phenoconversion-aware AI systems. LLMs may help identify relevant covariates and evidence distributed across clinical records, but they cannot establish the effect size of a covariate, infer an unknown interaction mechanism, or generate a validated dose adjustment when the underlying pharmacological relationship has not been characterized (Guevara et al., 2024).

The ability to translate such systems from research environments into routine clinical practice also depends on their generalizability (Finlayson et al., 2021). Models developed and validated within a specific healthcare system may not perform equally well in different clinical environments. Differences in EHR structure and documentation practices may be particularly important because phenoconversion-relevant information is often embedded in free-text clinical narratives. Variations in language, documentation practices, coding standards, prescribing patterns, patient demographics, and healthcare delivery models can all influence model performance. Pharmacogenomic diversity across populations further complicates this challenge, as allele frequencies and genotype-phenotype relationships may differ substantially among ancestry groups. As a result, models trained in a limited population may not generalize effectively to broader or more diverse patient cohorts. External validation across multiple institutions, countries, and patient populations would likely be essential.

Even systems that demonstrate adequate reliability and generalizability must ultimately be feasible to implement at scale. The computational requirements associated with large-scale deployment of LLM-based clinical decision-support systems may be substantial. A framework capable of continuously analyzing longitudinal EHR data, pharmacogenomic results, laboratory values, and clinical narratives across large patient populations would require considerable storage capacity, processing power, and maintenance. These costs may be manageable for large academic medical centers or healthcare networks but could represent a significant barrier for smaller institutions (Klang et al., 2024; Garcia-Agundez et al., 2025). Additional expenses for software development, cybersecurity, model updates, and validation must also be considered when evaluating implementation feasibility.

As these systems move closer to clinical deployment, regulatory considerations become equally important. AI-enabled clinical decision-support systems are increasingly subject to regulatory oversight. Determining the appropriate level of evidence required for approval, the responsibilities of developers and healthcare institutions, and the legal accountability for AI-assisted recommendations remain ongoing challenges. Regulatory frameworks will need to balance innovation with patient safety, ensuring that AI systems provide clinically meaningful benefits while maintaining appropriate standards of reliability and accountability.

Finally, these technical, scientific, and regulatory considerations intersect with broader ethical questions surrounding the use of LLMs in pharmacogenomic workflows. Patient privacy represents a major concern because these systems require access to highly sensitive genomic and clinical information. Transparency is equally important: clinicians and patients should be able to understand how recommendations are generated, while black-box approaches may reduce trust and complicate error investigation. Bias also merits attention. If training datasets fail to adequately represent diverse populations, prediction performance may vary across ancestry groups. Because pharmacogenomics already faces challenges related to population diversity, developers must ensure equitable performance across populations. Ultimately, clinicians must remain responsible for final decisions. AI tools should augment, rather than replace, professional judgment. Accordingly, LLM-based phenoconversion assessment should be viewed as a clinical decision-support tool rather than an autonomous decision-making system.

## Future directions

Future progress will first require stronger foundational evidence defining how genetic and non-genetic covariates influence enzyme and transporter activity, pharmacokinetics, and pharmacodynamics, both individually and in combination. Studies should quantify the magnitude, timing, and duration of these effects; establish validated measures of functional metabolic capacity; and determine when changes in metabolic activity produce clinically meaningful differences in drug exposure, efficacy, or toxicity. Without such evidence, neither LLM-assisted systems nor conventional model-informed precision-dosing approaches can generate sufficiently reliable and clinically validated drug-specific recommendations (Minichmayr et al., 2024; Nahid and Johnson, 2022; Scodellaro et al., 2026; Ali et al., 2025). Building on this pharmacological and pharmacometric foundation, future research should evaluate the ability of LLM-assisted systems to identify and temporally align phenoconversion-relevant information in real-world clinical records. Most current applications remain conceptual or exploratory, and their clinical value will need to be demonstrated through studies evaluating medication safety, therapeutic efficacy, adverse drug reactions, healthcare utilization, and patient outcomes. Establishing standardized phenoconversion frameworks and clinically validated endpoints will be essential for comparing results across studies and healthcare systems.

Another promising direction is the integration of pharmacogenomic and phenoconversion-related information into established dynamic modelling and model-informed precision-dosing frameworks. Future platforms may integrate pharmacogenomic results with medication exposure, laboratory data, inflammatory biomarkers, organ function, lifestyle factors, and other determinants of enzyme activity to generate continuously updated estimates of functional metabolic capacity. The combination of LLMs with pharmacokinetic modeling, knowledge graphs, and mechanistic pharmacology could enable probabilistic assessments of drug-metabolizing activity that more accurately reflect the patient’s current biological state. In this combined framework, LLMs could support the retrieval, structuring, temporal contextualization, and communication of relevant clinical information, including factors associated with dynamic drug-response phenotypes for drugs without actionable pharmacogenomic recommendations. Quantitative predictions should remain the responsibility of appropriately validated pharmacometric or mechanistic models. This division of functions is safer and more scientifically defensible than asking an LLM to infer metabolic capacity or dose requirements directly from clinical text. Integration with multimodal data sources will further expand the potential of these approaches. In addition to electronic health record narratives, future systems may incorporate genomic data, transcriptomic and metabolomic profiles, therapeutic drug monitoring results, wearable sensor data, and imaging information. Such multimodal frameworks could provide a more comprehensive representation of the factors influencing drug response and help bridge the gap between genotype and phenotype. In parallel, integration with existing pharmacogenomic infrastructures, including CPIC, DPWG, PharmGKB, PharmVar, ClinVar, and ClinGen, will be critical to ensure that AI-supported information retrieval and decision-support outputs remain aligned with current evidence, nomenclature, and prescribing guidelines.

Importantly, LLMs may also provide a powerful tool for retrospective pharmacogenomic discovery. By analyzing large-scale electronic health records, adverse event databases, and longitudinal clinical data, these systems could identify potential, previously unrecognized phenoconversion events associated with adverse drug reactions, treatment failures, or unexpected variability in therapeutic response. Such analyses could generate hypotheses regarding previously unrecognized drug–drug–gene and drug–gene–environment interactions and identify candidate factors for subsequent pharmacoepidemiological, pharmacokinetic, or prospective evaluation. Retrospective real-world evidence could therefore complement prospective studies and contribute to a more complete understanding of drug-response variability, both for drugs with established actionable pharmacogenomic recommendations and for those without a validated actionable gene–drug relationship.

Ultimately, the most transformative application of LLMs may not be limited to detecting phenoconversion at the individual patient level but also to continuously learning from large patient populations. By systematically identifying genotype-phenotype discrepancies and linking them to clinical outcomes, AI-enabled systems could refine pharmacogenomic knowledge over time, generate new hypotheses, and support the iterative improvement of prescribing recommendations. In this way, LLMs may help drive the evolution of pharmacogenomics from a largely static, genotype-centered discipline toward a dynamic and continuously updated model of personalized therapy, in which both genetic and non-genetic determinants of drug response are considered to optimize treatment efficacy and safety.

## Conclusion

Phenoconversion complicates the clinical interpretation of pharmacogenomic results because current drug-metabolizing capacity may be modified by medications, inflammation, organ dysfunction, physiological changes, and environmental exposures that are not captured by genotype alone. Genotype-predicted phenotypes may therefore differ from functional metabolic activity at the time of treatment.

LLMs could support phenoconversion-aware clinical decision making by retrieving, structuring, and temporally contextualizing relevant information from electronic health records. However, LLMs cannot independently establish that phenoconversion has occurred or quantify its effect on metabolic activity, drug exposure, dose requirements, or clinical response. These functions require validated phenotypic measurements and drug-specific pharmacometric or mechanistic models.

The clinical value of this approach will depend on the quality of the underlying evidence, the completeness of clinical data, external validation, uncertainty reporting, transparent documentation, and continued human oversight. Properly integrated with established pharmacogenomic and precision-dosing frameworks, LLMs may help move clinical interpretation from static genotype assignment toward more dynamic and context-aware assessment.

## Data Availability

The original contributions presented in the study are included in the article/supplementary material, further inquiries can be directed to the corresponding author.
